# Impact of Work-From-Home Human Resource Practices on the Performance of Online Teaching Faculty During Coronavirus Disease 2019

**DOI:** 10.3389/fpsyg.2021.740644

**Published:** 2021-10-28

**Authors:** Huda Irshad, Khawaja Muhammad Umar, Mahmood Rehmani, Munnawar Naz Khokhar, Naveed Anwar, Ayaz Qaiser, Rana Tahir Naveed

**Affiliations:** ^1^Department of Business Administration, University of Sialkot, Sialkot, Pakistan; ^2^Department of Management Sciences, COMSATS University, Islamabad, Pakistan; ^3^Department of Management Sciences, Shaheed Zulfiqar Ali Bhutto Institute of Science and Technology, Larkana, Pakistan; ^4^Department of Economics and Business Administration, Division of Management and Administrative Sciences, University of Education, Lahore, Pakistan

**Keywords:** work-from-home, HR practices, online teaching faculty, employee performance, COVID-19

## Abstract

The purpose of this study was to investigate the impact of work-from-home (WFH) human resource (HR) practices on the performance of faculty under the drastic circumstances of coronavirus disease 2019 (COVID-19). The population of the study included faculty members of the higher education institutions in Sialkot, Pakistan. The study filled the gap of scarce literature on the impact of various HR practices by HR officials while working from home during lockdown observed to reduce the spread of COVID-19. Based on reinforcement theory, this article proves that there is a significant relationship between HR practices (including training, performance appraisal, career planning, employee participation, job definition, compensation, and selection) and faculty performance. It also proves that there is a strong positive relationship between the two variables. The findings of this study provide a blueprint to improve HR practices for high performance by faculty in the higher education sector during WFH settings.

## Introduction

Coronavirus disease 2019 (COVID-19) is a contagious respiratory and vascular disease caused by severe acute respiratory syndrome coronavirus 2 (SARS-CoV-2). While the pandemic has cost human lives, it has disrupted the economy and social aspects of life. According to the survey results of Marinoni et al. (2020), higher education institutions (HEIs) around the world are suffering due to the COVID-19 crisis in one way or the other. The regional analysis shows that Asia is at the brink of a growing risk of inequality among HEIs due to lack of access to technology and financial downfall. Qamar and Bawany (2021) state that Pakistan followed the footsteps of global educational institutions and switched to an online mode of learning, but this sudden change was not welcomed by all educationists and students. Rehman (2020) states the reason Pakistan was among the most affected countries as online education was an alien method for the people. The majority of the workforce consists of human capital and the need for hours stated a reformation in the workplace dynamics and that is how COVID-19 led to normalizing working from home. There is evidence that human resource (HR) practices positively affect employee performance and in the context of Pakistan studies (Bashir and Khattak, 2008; Shahzad et al., 2008; Bowra et al., 2012; Hameed et al., 2019, 2020; Khan et al., 2021b). Several HR practices can be linked with employee performance. Aycan et al. (2000) have called Pakistan an under-researched country in the context of HR management (HRM) practices as it is a developing country. During COVID-19, companies adopted the work-from-home (WFH) culture to survive, and it not only enhances the productivity of employees but also offers greater flexibility in work arrangements and creates better work-life balance (Dizaho et al., 2017).

This study attempts to fill the research gap in a developing country like Pakistan by finding a relationship among HR practices such as recruitment and selection, training and development, career management, and so on (Bashir and Khattak, 2008). Another study by Khalid et al. (2014) studied HR practices as career planning, performance appraisal, training, compensation, and employee participation concerning employee performance but research was limited to one city and public organizations. Any performance of an organization depends on the performance of its employees, which is measured by the HRM. The performance of an employee is what he/she does or does not do (Shahzadi et al., 2014; Sheikh et al., 2017; Islam et al., 2019). In the case of institutes, we turn employee performance into faculty performance as a variable. This study aims to analyze the link between seven HR practices and faculty performances in HEIs. Researchers have not conducted any study in institutes of Sialkot, Punjab, Pakistan to measure faculty performance during online classes. HR officials have implemented HR practices from home and faculty members have adhered to them working from home as well. The objective of the current study is to focus on the impact of WFH HR practices on faculty performance and whether these practices are linked with faculty performance or not. The results of the study significantly contribute to the previous literature and offer suggestions to HR managers for making the practices better in these difficult times.

### Literature Review and Hypothesis Development

Armstrong (2006) defined HRM as a strategic and coherent approach to the management of the most valued assets of an organization, the people working there who individually and collectively contribute to the achievement of its objectives. The sudden transition of the education system to the online medium caused a stir. As much as students have faced troubles during learning, the digital rules and punctualities, so have the online teaching faculty found many challenges. Schools and colleges have principals and the level of management is slightly on a smaller scale than universities. Educational institutes may or may not have an integrated HR department but they perform practices such as planning, organizing, and managing the personnel. The higher management or the HR manager is responsible for setting the mission and the goals, motivating the employees, deciding on the best guidelines, and so on. The best HR practices include recruitment and selection, training and development, transparency, employee benefits, employee incentives, compensation and evaluations, compliance, and termination. As the foremost orders by the government strictly imposed social distancing, the challenge began to keep the organizations running smoothly, which meant a world without direct interactions.

The outbreak of COVID-19 has taught us that change is inevitable (Islam et al., 2020). The first preventative step suggested by WHO was social distancing. Every country closed schools, colleges, and universities. Moreover, the government canceled entrance tests, examinations, classes, and internships. It took students as well as the faculty by storm as adapting to the digitized education system was not easy. Change requires time; however, the pandemic caused the education sector in India to grow. Online education has proved to be a salvation for the students and teachers. They assigned work to students *via* the internet and delivered lectures through live video conferencing using applications like Zoom, Google meets Facebook, YouTube, and Skype. There are WhatsApp groups that help keep students, teachers, and even guardians connected and aware of the class schedules. Online learning is the best solution and is surely better than not getting to learn anything (Jena, 2020).

To better understand the impact of HR practices on faculty commitment, an empirical study has been conducted by Rahiman et al. (2018) to analyze demographic factors that have an impact on HR practices, the influence of explanatory variables on organizational commitment, the relationship between HR practices and organizational commitment, and, in addition, to suggest improvements in HR practices and commitment level. Quality of teaching staff and faculty commitment need to be restructured, which will, in turn, increase the quality of education and student satisfaction. It is only fair to say if the organization provides staff with the best training and environment, it directly affects the results of students. For stability and growth, every organization must have an integrated HR system. HR is the backbone of any organization and it helps carry out all organizational operations. HRM practices, namely, training and development, performance appraisals, teamwork, HR planning, compensations, safety, and health, help to enhance the performance of an employee by creating a psychological link with the organization that prevents him/her from leaving. Organizational commitment in higher education is necessary as teachers are not only facilitators for students but they thrive for their personal development as well.

Work-from-home has its pros and cons for teachers. If both the teacher and school go through it responsibly and both parties understand the conditions that occur, they can carry out WFH effectively. Even though the distance exists, it is important to maximize communications to avoid unnecessary issues (Purwanto et al., 2020). A successful performance management system helps in evaluating and improving both company and individual performance. The organization must not avoid performance evaluation, as it will result in poor business decisions. Employee recruitment and selection were being discouraged due to the pandemic especially in private organizations to avoid unnecessary spending. Managing employee performance was found to be challenging. Employees were unable to set realistic goals and even failed to achieve previously set goals. Monitoring performance was problematic and coordinating activities seemed too tedious. This affected compensation management, as the company no longer offered incentives. Some organizations got too far by asking their employees to take leave without pay and some were thinking about pay cuts. Private organizations suffered the most effects of the pandemic. When a company stops rewarding the employees, it destroys employee motivation. HRM practices are not immune to COVID-19. Employee recruitment and selection became more difficult in the time when employees voluntarily or involuntarily leave organizations. Employee training programs decreased and this challenging performance management, as monitoring from home is not easy. Jobs in public organizations are more secure than in private organizations and organizations should adopt electronic HRM (EHRM) and review HR policies (Mwita, 2020).

The COVID-19 pandemic has no duration and it forced rapid improvization and adoption of online teaching methods in educational institutes (Hou et al., 2021; Islam et al., 2021; Khan et al., 2021a). They used the approach based on synchronous and asynchronous learning. Synchronous learning is online or distance learning that is based on real-time interactions between students and facilitators. On the other hand, asynchronous learning occurs through online platforms without real-time interactions. Now that online education is taking place in the sector, online instructors need to take the role of facilitators and not just provide education but also enhance the learning of students. Online teaching methods, such as the open-source learning management system (LMS), used a free version of Zoom in different modules of Singapore. The adoption of LMS by HEIs to support student learning goes back to the late 1990s. They developed LMS platforms around three interrelated functions: (1) provision and organization of content, (2) course management including attendance, assessment, grade management, and announcements, and (3) communication tools. Online teaching requires three types of interrelated preparation. First, there is a process of selection, presentation, and grouping of resources. Second, there is planning required for each hour of online delivery and third, teaching online is much more tiring than classrooms. It involves managing multiple cues from students including engaging in voice-based discussions while observing and managing public and private chat rooms.

Lecturers working from home face many challenges as the virtual classrooms introduced a new form of encounter with family members especially children or pets. Online-only blended learning is still an unusual form of the classroom. The purpose of this transition is to provide a platform equally for every student in any geographical location and even if he or she is exercising quarantine. Online makes it difficult to connect for students. The shift to online teaching forms the role of the instructor from teacher to guide, facilitator, coordinator, challenger, stimulator, encourager, or conductor. Students require more online self-discipline. COVID-19 is forcing deeper adoption of the LMS system and the development of a curation-oriented learning paradigm. For module development, the process of curation takes two forms: curating content to support learning outcomes and curating student experiences. The latter is important as it reflects a balance between students and lecturers. In online encounters, those involved must work harder to read non-verbal or social cues including gestures, expressions and tone, and pitch of the voice. Undoubtedly, the transition to online learning has been very tiring. As nothing can replace proximate learning, the modules stated that students should be encouraged to discuss and engage in sessions (Bryson and Andres, 2020).

According to the ***reinforcement theory***, behavior is a function of its consequence. The consequences that immediately follow behavior and increase the probability to repeat a behavior are ***reinforcers***. The biggest HR challenges of the year 2020 were focusing on employees and communicating often. It is human psychology when efforts go unappreciated the person is automatically demoralized, which results in the prevention of further out-of-the-mile hard work. Getting used to the online education transition has been reckless to the faculty and they deserve sufficient motivation even if it is a single appreciation email. According to Skinner (1958), people will most likely engage in desired behaviors if they receive a reward for doing so. Managers can easily influence employee behaviors by using positive reinforcers for actions that help the organization achieve its goal, which in this case is maximum faculty performance. This will equilibrate the effects of HR practices from home on online teaching faculty.

### Relationship Between HR Practices and Online Teaching Faculty Performance

**Figure 1:**
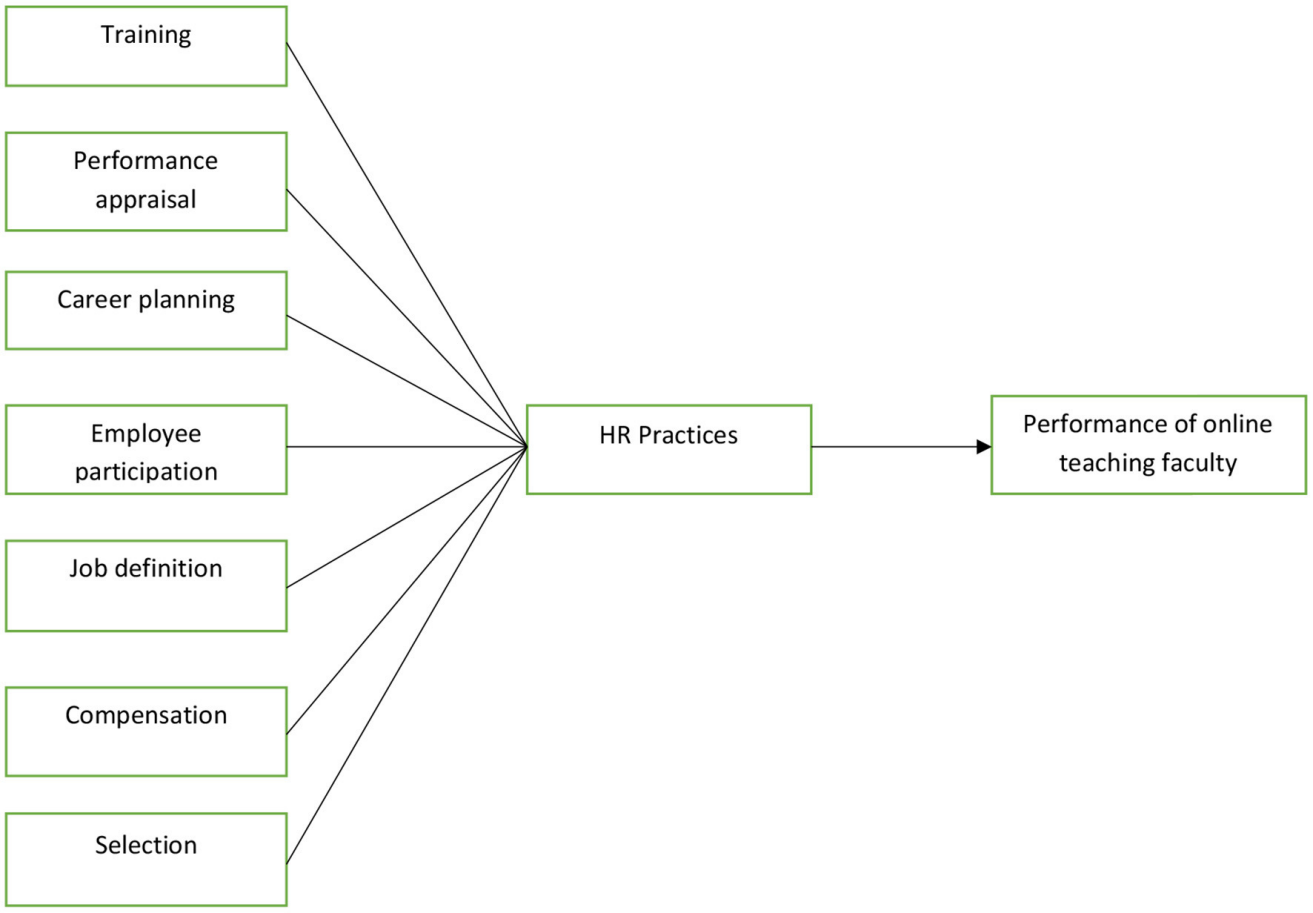
Theoretical framework.

Teachers and lecturers can deliver lectures through technology. WFH provides flexibility in completing work. This cuts down transportation costs and saves time. Even if the factor of stress occurs, the faculty can easily find comfort in the atmosphere of his/her home, and this way productivity increases. With this comes job satisfaction, which certainly increases loyalty to the organization for faculty. On the other hand, loss of work motivation working from home can severely affect the performance of faculty. Moreover, due to the daily increased use of the internet and electricity, the individual has to bear the cost of working from home (Purwanto et al., 2020). Employers, as well as HR managers, have been compelled to redefine HR roles and bring about best practices. HR plays an important role to bring the concept of “people connect” to light and create a bond with the people at the organization. It is up to HR to make the home of an employee a substitute for the workplace. The relative success of WFH is one of the achievements worldwide in this disaster. HR is responsible for motivating, inspiring, training, and developing the people, normalizing usage of technology to efficiently work and manage work relationships according to the changing scenarios (Kaushik and Guleria, 2020). There is research evidence that a strong positive relationship exists between HR practices and employee performance as it can be seen in Figure 1 (Bashir and Khattak, 2008; Shahzad et al., 2008; Bowra et al., 2012; Khalid et al., 2014). Each construct including seven HR practices and faculty performance has been defined in Table 1. Hence, we hypothesize the following:

H1: There is a significant positive effect of WFH HR practices on the performance of online teaching faculty.

**Table 1:** Constructs defined.

Training	“A learning process where people learn knowledge, attitudes, concept, and skills to achieve organizational goals” (Ripley, 2002)
Performance appraisal	“The process by which superiors evaluate the performance of employees for determining training needs or promotions” (Grote and Grote, 2011)
Career planning	“Process of targeting career development and implementation of strategies, self-assessment, analysis and evaluation of results” (Antoniu, 2010)
Employee participation	“Participation of non-managerial employees in decision making” (Kapur, 2018)
Job definition	“A list of job duties, responsibilities, reporting relationships, working conditions, and supervisory responsibilities” (Valenzi and Dessler, 1978)
Compensation	“ A combination of non-financial and financial remuneration given to employees in return for performance by the employer as salary, bonus etc.” (Osibanjo et al., 2014)
Selection	“The process of choosing the most suitable person from a pool of candidates, within or outside the organization” (Koontz, 2010)
Faculty performance	“The degree to which a faculty member has achieved goals of teaching or service at the organization” (Kurz et al., 1989)

### Faculty Performance

Performance is what an organization hires an employee for. It is the outcome of a task for an individual (Shahzadi et al., 2014). Employee or job performance is related to organizational goals and it is the result of the work of an employee. The aggregated value to an organization of the set of behaviors that employees contribute to fulfilling organizational goals is employee performance (Borman and Motowidlo, 1993). In educational institutes, employees are called faculty members and so the dependent variable of this study is faculty performance. Faculty performance deals with outcomes and accomplishments at work (Anitha, 2014). Moreover, the behavior of an employee is important for defining his/her performance (Aguinis, 2009).

### HR Practices

Effective HR management practices can be the main factor for the success of a firm (Stavrou-Costea, 2005). HR practices help to improve business performance including employee productivity (Lee and Lee, 2007). HR also provides an orientation to the employees allowing them to understand the organizational culture, which encourages performance. For faculty working from home, HR proves to be a ray of light. HR practices significantly affect academic staff performance (Hashim et al., 2017; Shahzad et al., 2020). Many researchers have studied the relationship between employee performance and HR practices but they confined the study to developed countries (Aycan et al., 2000). Few pieces of research in Pakistan found a significant relation between HR practices and employee performance but the study was limited to public sector universities (Shahzad et al., 2008).

### Training

Firms can provide training and development to employees to increase their performance levels. An employee is satisfied only when he/she feels eligible for performing his/her job through training programs (Elnaga and Imran, 2013). Training allows employees to have a better understanding of workplace ethics and their responsibilities. This practice is considered best as it brings about the desired change in employee performance (Delaney and Huselid, 1996). May it be information or new skills training impacts employee performance, we formulate the following hypothesis to find out whether the HR practice training and faculty performance has a relationship.

H2: There is a significant relationship between training and the performance of online teaching faculty.

### Performance Appraisal

The appraisal of employees may be performance-based and attendance-based. The HR monitors and evaluates performance or attendance by using ranking scales. Appraisals are the process of evaluating how employees perform at work and the responsibilities assigned to them in comparison to the objectives that have been set for them. According to the job descriptions, HR measures the present performance concerning the expected performance. If the performance needs improvement, HR manages it. Appraisal-based information is helpful in the training process. An important aspect of appraisal is employee motivation, which ensures better performance (Singh, 2004). We can hypothesize that

H3: There is a significant relationship between performance appraisal and the performance of online teaching faculty.

### Career Planning

Selecting career goals and paths to these goals allows employees to understand personal and organizational goals with clarity. This encourages employees to plan accordingly on how to reach their full capacity. As a result, the employee finds motivation and satisfaction, which enhances performance (Beardwell and Claydon, 2007). There is a relationship between career planning and performance, we hypothesize the following to test this.

H4: There is a significant relationship between career planning and the performance of online teaching faculty.

### Employee Participation

Participation has statistically significant effects on the performance, satisfaction, and productivity of an employee (Wagner, 1994). Maslow's theory of motivation states clearly that the need to earn money is not the only motive but being involved in decision-making and problem-solving provides a greater sense of belonging and motivates to perform better under the job description. We develop a hypothesis to find out the relationship between the HR practice of employee participation and faculty performance.

H5: There is a significant relationship between employee participation and the performance of online teaching faculty.

### Job Definition

If employees are involved in defining their job and are well-acquainted with the requirements, it increases their motivation level. Defining a job means forming a job description and job specification. An overview of duties, responsibilities, and functions of a specific job is the job description. The qualifications and skills required for that job are the job specifications. If the organization confines job definition to the set standards, it prevents the employee to perform at his/her full potential (Singh, 2004). During online teaching, job definition helps to enhance the performance of faculty or not we formulate the following hypotheses to evaluate this.

H6: There is a significant relationship between job definition and the performance of online teaching faculty.

### Compensation

Incentives and bonuses comprise compensation and benefits for the employees. As each HR practice is somewhat connected, performance appraisal backs up the compensation. As the supporting theory suggests reinforcing employee performance has fruitful results. Incentive-based compensation has an impact on firm performance (Milkovich et al., 1996). Compensation can help in maintaining the productivity of the employees or else they tend to leave the organization searching for better opportunities where they are rewarded better (Darma and Supriyanto, 2017).

H7: There is a significant relationship between compensation and performance of online teaching faculty.

### Selection

The backbone of HR practices in recruitment is selection. Out of a pool of candidates through interviews, employees are hired. The better the selection system, the higher the expectations of performance are. It falls upon the interviewer to select carefully the right person for the respective job or it can cost the organization a lot of time and resources. Firm performance relates to selection positively (Terpstra and Rozell, 1993). To confirm the relationship of selection with the performance of online teaching faculty, we form the following hypotheses.

H8: There is a significant relationship between selection and performance of online teaching faculty.

## Materials and Methods

Higher education institutes in Sialkot, Punjab, Pakistan, were selected to examine the relationship between HR practices and faculty performance. Through Higher Education Commission (HEC) Pakistan's website, we found out there were only four such institutes in Sialkot. The total population consisted of 709 faculty members employed at the institutes. The survey questionnaire method was used to collect data and through emails, the target respondents were reached out. The questionnaire consisted of instruments formed by Singh (2004) to measure HR practices. To measure the performance of online teaching faculty, the scale developed by Pradhan and Jena (2017) is used. Due to the strict policies of lockdown, we were not allowed to visit the universities, so an online survey aided us favorably. We used convenience sampling to overcome the COVID-19 constraints and 179 responses were collected in 3 months.

### Independent Variable

Human resource practices consist of seven indicators: Training, performance appraisal, career planning, employee participation, job definition, compensation, and selection. We used the Likert scale to measure each indicator. Each scale was a five-point scale with “1” = strongly disagree to “5” = strongly agree. The respondents were asked to indicate their opinion by the five-scale. In the questionnaire, we wrote the scale as follows: “5” = strongly agree, “4” = agree, “3” = neutral, “2” = disagree, and “1” = strongly disagree. This helped respondents understand what it would mean exactly to choose the different rating scales. Training has six items; respondents chose the scale to denote the need for training. Performance appraisal has seven items; we asked respondents to indicate the extent to which performance is evaluated. Employee participation has three items; we expected respondents to express the level of liberty to speak in managerial decisions. Job definition has four items; we asked respondents to indicate the extent of the definition of a job. Compensation has five items; we required respondents to choose the scale expressing the relation of performance with compensation. The selection has four items; we asked respondents to indicate the importance of selection tools. HR practices variable has 36 indicators.

### Dependent Variable

**Figure 2:**
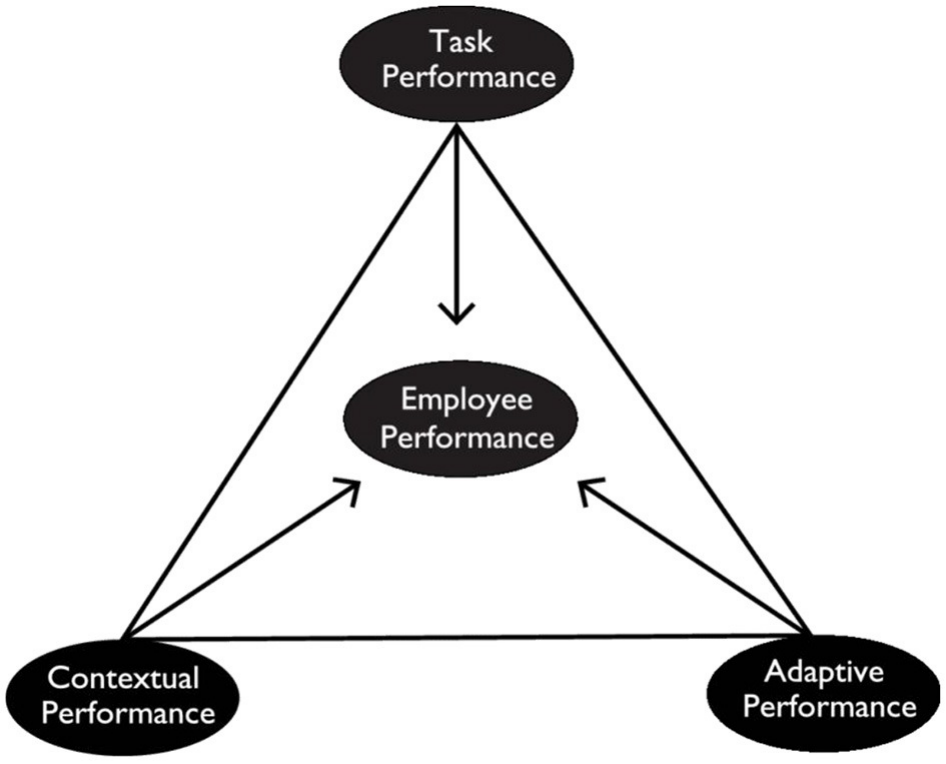
The Triarchy model of employee performance. Source: Pradhan et al. (2016).

We measured faculty performance using the tool for employee performance (Figure 2). It consists of the triarchy model of employee performance suggested by Pradhan and Jena (2017). Task performance, adaptive performance, and contextual performance comprise employee performance. Faculty performance has 22 indicators.

### Data Collection and Analysis

We have used International Business Machines Statistical Package for Social Sciences (IBM SPSS) 25.0 (x64) for conducting descriptive, regression, and correlation analysis. The demographic factors include age, gender, and work experience. The following tables provide the statistics of demographics:

Table 2 shows the majority of respondents are from the age bracket of 20–30 (129), which is 72.1% of the data. Only 36 respondents are of the ages 30–40 and this is 20.1% of the sample. Remaining respondents belong to the ages 40–50 (3.9%), 50–60 (3.4%), or 60 and above (0.6%). The majority of the respondents are female. The received responses from women are 78.2% (140), while the remaining 21.8% (39) are from men. There are 130 respondents out of 179 having work experience of fewer than 5 years. This counts for 72.6% of the data. Only 40 respondents have 5–15 years of experience (22.3%), while the remaining 5% have 15 years or more of work experience.

**Table 2:** Descriptive analysis.

**S.no**	**Demographic variable**	**Demographic characteristics**	**Frequency**	**Percentage**
1	**Age**			
		20–30	129	72.1
		30–40	36	20.1
		40–50	7	3.9
		50–60	6	3.4
		60 and above	1	0.6
		Total	179	100.0
2	**Gender**			
		Female	140	78.2
		Male	39	21.8
		Total	179	100.0
3	**Work Experience**			
		Less than 5 years	130	72.6
		5–15 years	40	22.3
		15 years or more	9	5.0
		Total	179	100.0

The reliability analysis deals with Cronbach's alpha and according to the general rule of thumb, it should be 0.70 or above. Table 3 shows the value of Cronbach's alpha for HR practices collectively and each indicator. HR practices (α = 0.966), training (α = 0.867), performance appraisal (α = 0.911), career planning (α = 0.888), employee participation (α = 0.802), job definition (α = 0.815), compensation (α = 0.800), selection (α = 0.781), and faculty performance (α = 0.940). The variables have high reliability and are acceptable for further analysis.

**Table 3:** Reliability statistics.

**Variables**	**Cronbach's alpha**	**N of Items**
Training	0.867	6
Performance appraisal	0.911	7
Career planning	0.888	7
Employee participation	0.802	3
Job definition	0.815	4
Compensation	0.800	5
Selection	0.781	4
HR practices	0.966	36
Faculty performance	0.940	22

The following table sets out the descriptive statistics in terms of means and SDs. The values of SD in Table 4 are higher than the mean of all variables. This means the variation in data is in a large range.

**Table 4:** Descriptive statistics.

	**Mean**	**SD**	***N***
Faculty performance	3.9010	0.70560	179
Training	3.7225	0.83836	179
Performance appraisal	3.7749	0.84636	179
Career planning	3.6824	0.80891	179
Employee participation	3.7002	0.90711	179
Job definition	3.7318	0.84760	179
Compensation	3.6335	0.82120	179
Selection	3.6103	0.85995	179

The table of correlation shows a significance at 0.01 level for each variable, which makes us accept the hypothesis. The values are statistically significant. We accept that there is a relationship between HR practices and the performance of online teaching faculty.

Based on the correlation coefficient, we conclude that there is a strong positive impact of WFH HR practices on the performance of online teaching faculty. A positive correlation means there is a direct relationship between the two variables. If the independent variable increases, the dependent variable will increase too.

## Results, Discussions, and Conclusions

The findings of this study are in communion with existing literature that there is a positive relationship between HR practices and performance (Shahzad et al., 2008; Akhter et al., 2013; Ali et al., 2020). Based on correlation results, we accept H2, H3, H4, H5, H6, H7, and H8 suggesting that training, performance appraisal, career planning, employee participation, job definition, compensation, and selection, respectively, have a statistically significant relationship with faculty performance in HEIs at Sialkot. Furthermore, the correlation coefficient shows the existence of a strong positive relationship between all HR practices understudy and faculty performance. This fills the literature gap by studying various HR practices in the educational context during COVID-19. As employee performance is measured by the HR management of any organization, so there is not much room for biases in the self-reported data of the study. As evident from Tables 5, 6, the HR practices individually and as a whole have a strong relationship (*p* ≤ 0.01) with faculty performance. So, we accept H1 and fulfill the objective of this study to find the impact of HR practices on the performance of online teaching faculty. Table 7 consists of each hypothesis and its decision.

**Table 5:** Correlation analysis.

		**Training**	**Performance appraisal**	**Career planning**	**Employee participation**	**Job definition**	**Compensation**	**Selection**	**Faculty performance**
Training	Pearson Correlation	1	0.786^**^	0.667^**^	0.554^**^	0.458^**^	0.535^**^	0.583^**^	0.461^**^
	Sig. (2-tailed)		0.000	0.000	0.000	0.000	0.000	0.000	0.000
	*N*	179	179	179	179	179	179	179	179
Performance appraisal	Pearson Correlation	0.786^**^	1	0.791^**^	0.666^**^	0.665^**^	0.664^**^	0.746^**^	0.513^**^
	Sig. (2-tailed)	0.000		0.000	0.000	0.000	0.000	0.000	0.000
	*N*	179	179	179	179	179	179	179	179
Career planning	Pearson Correlation	0.667^**^	0.791^**^	1	0.758^**^	0.700^**^	0.711^**^	0.776^**^	0.554^**^
	Sig. (2-tailed)	0.000	0.000		0.000	0.000	0.000	0.000	0.000
	*N*	179	179	179	179	179	179	179	179
Employee participation	Pearson Correlation	0.554^**^	0.666^**^	0.758^**^	1	0.728^**^	0.751^**^	0.741^**^	0.458^**^
	Sig. (2-tailed)	0.000	0.000	0.000		0.000	0.000	0.000	0.000
	*N*	179	179	179	179	179	179	179	179
Job definition	Pearson Correlation	0.458^**^	0.665^**^	0.700^**^	0.728^**^	1	0.703^**^	0.707^**^	0.407^**^
	Sig. (2-tailed)	0.000	0.000	0.000	0.000		0.000	0.000	0.000
	*N*	179	179	179	179	179	179	179	179
Compensation	Pearson Correlation	0.535^**^	0.664^**^	0.711^**^	0.751^**^	0.703^**^	1	0.756^**^	0.466^**^
	Sig. (2-tailed)	0.000	0.000	0.000	0.000	0.000		0.000	0.000
	*N*	179	179	179	179	179	179	179	179
Selection	Pearson Correlation	0.583^**^	0.746^**^	0.776^**^	0.741^**^	0.707^**^	0.756^**^	1	0.514^**^
	Sig. (2-tailed)	0.000	0.000	0.000	0.000	0.000	0.000		0.000
	*N*	179	179	179	179	179	179	179	179
Faculty performance	Pearson Correlation	0.461^**^	0.513^**^	0.554^**^	0.458^**^	0.407^**^	0.466^**^	0.514^**^	1
	Sig. (2-tailed)	0.000	0.000	0.000	0.000	0.000	0.000	0.000	
	*N*	179	179	179	179	179	179	179	179

** *Correlation is significant at the 0.01 level (2-tailed)*.

**Table 6:** Coefficients^a^.

**Model**		**Unstandardized Coefficients**		**Standardized Coefficients**	**t**	**Sig**.
	**B**	**Std. Error**	**Beta**		
1	(Constant)	1.846	0.227		8.117	0.000
	HR Practices	0.555	0.060	0.569	9.203	0.000

a *Dependent Variable: Faculty performance*.

**Table 7:** Hypothesis decisions.

H1: There is a significant positive effect of work-from-home human resource practices on the performance of online teaching faculty	Accepted
H2: There is a significant relationship between training and performance of online teaching faculty	Accepted
H3: There is a significant relationship between performance appraisal and the performance of online teaching faculty	Accepted
H4: There is a significant relationship between career planning and the performance of online teaching faculty	Accepted
H5: There is a significant relationship between employee participation and the performance of online teaching faculty	Accepted
H6: There is a significant relationship between job definition and performance of online teaching faculty	Accepted
H7: There is a significant relationship between compensation and performance of online teaching faculty	Accepted
H8: There is a significant relationship between selection and performance of online teaching faculty	Accepted

### Research Implications

Although correlation analysis shows significant values, HR managers should encourage employee participation in decision-making and make idea-sharing comfortable. This will in turn instill modern HR practices and improve their understanding of these practices. The findings of the study prove how the HR of an organization can result in a competitive advantage. A high-performing employee can lead to organizational success. Given the conditions of WFH, it is best to revise compensation plans as Pakistan has a low GDP and it is essential to prevent large-scale unemployment. It is not only a pandemic but a new reality for organizations and the only way is to strategically comply. In short, the findings of this study provide a blueprint to enhance HR practices for higher employee performance.

### Limitations and Suggestions

The research context is limited to HEIs in Sialkot and due to limited reachability sample size could not be large. For future research, more cities should be included in the target population to test the linkages between HR practices and faculty performance. Moreover, the addition of a mediator as transformational leadership might help explore other factors that strengthen the relationship under study. Data collection through online surveys may have caused a hurdle for respondents who lacked accessibility, so future research should use personally administered questionnaires and interviews as well. The majority of the respondents were females (78.2%) while the data collection was intended to show results from both genders. Finally, respondents should be from both public and private universities to expand the study. Improving the learning outcomes and quality of teachers is necessary and not just because of the pandemic. In education, technology is the new medium to ensure the literacy rate getting better in Pakistan.

## Data Availability Statement

The original contributions presented in the study are included in the article/supplementary material, further inquiries can be directed to the corresponding author/s.

## Author Contributions

HI conceptualized the research topic. KU helped with the data collection. The final draft was made by HI. All this is done under the supervision of the very informative and supportive MR. Visualization and validation were done by NA. Proofreading has been done by MK while AQ and RN have helped make revisions in the manuscript. All authors contributed to the article and approved the submitted version.

## Conflict of Interest

The authors declare that the research was conducted in the absence of any commercial or financial relationships that could be construed as a potential conflict of interest.

## Publisher's Note

All claims expressed in this article are solely those of the authors and do not necessarily represent those of their affiliated organizations, or those of the publisher, the editors and the reviewers. Any product that may be evaluated in this article, or claim that may be made by its manufacturer, is not guaranteed or endorsed by the publisher.
